# Inter- and Intra-Hemispheric Age-Related Remodeling in Visuo-Spatial Working Memory

**DOI:** 10.3389/fnagi.2021.807907

**Published:** 2022-01-17

**Authors:** Chiara F. Tagliabue, Greta Varesio, Veronica Mazza

**Affiliations:** Center for Mind/Brain Sciences (CIMeC), University of Trento, Trento, Italy

**Keywords:** visuo-spatial working memory, neurocognitive aging, electrophysiology, Hemispheric Asymmetry Reduction in Older Adults (HAROLD), Posterior-Anterior Shift in Aging (PASA)

## Abstract

Electroencephalography (EEG) studies investigating visuo-spatial working memory (vWM) in aging typically adopt an event-related potential (ERP) analysis approach that has shed light on the age-related changes during item retention and retrieval. However, this approach does not fully enable a detailed description of the time course of the neural dynamics related to aging. The most frequent age-related changes in brain activity have been described by two influential models of neurocognitive aging, the Hemispheric Asymmetry Reduction in Older Adults (HAROLD) and the Posterior-Anterior Shift in Aging (PASA). These models posit that older adults tend to recruit additional brain areas (bilateral as predicted by HAROLD and anterior as predicted by PASA) when performing several cognitive tasks. We tested younger (*N* = 36) and older adults (*N* = 35) in a typical vWM task (delayed match-to-sample) where participants have to retain items and then compare them to a sample. Through a data-driven whole scalp EEG analysis we aimed at characterizing the temporal dynamics of the age-related activations predicted by the two models, both across and within different stages of stimulus processing. Behaviorally, younger outperformed older adults. The EEG analysis showed that older adults engaged supplementary bilateral posterior and frontal sites when processing different levels of memory load, in line with both HAROLD and PASA-like activations. Interestingly, these age-related supplementary activations dynamically developed over time. Indeed, they varied across different stages of stimulus processing, with HAROLD-like modulations being mainly present during item retention, and PASA-like activity during both retention and retrieval. Overall, the present results suggest that age-related neural changes are not a phenomenon indiscriminately present throughout all levels of cognitive processing.

## Introduction

A gradual decrement in visuo-spatial working memory (vWM) marks the progression of physiological aging (Salthouse et al., 1991; Jenkins et al., 2000; Park et al., 2002), since this cognitive ability is implicated in several everyday activities (Davies and Logie, 1993). The deficit in vWM has been observed in terms of behavioral performance across different tasks. For instance, older adults exhibit a decrease in their visuo-spatial span for block sequences and patterns (e.g., Beigneux et al., 2007), perform worse than younger in the n-back task (e.g., Mattay et al., 2006), and retain fewer relevant items when presented among distractors (e.g., Jost et al., 2011).

The age-related vWM decrease has also been evaluated at the neural level through different imaging techniques (e.g., EEG, fMRI, MEG; for reviews: Rajah and D’Esposito, 2005; Tagliabue and Mazza, 2021). For instance, several EEG studies have provided robust evidence for the neural correlates of age-related vWM decline. The lateralized delayed match-to-sample (DMTS) judgment is a typical task used in EEG studies to investigate vWM in the aging population (e.g., Jost et al., 2011; Sander et al., 2011; Störmer et al., 2013; Ko et al., 2014; Schwarzkopp et al., 2016; Tagliabue et al., 2019), although it was originally developed in animal research investigating vWM ability (e.g., Herman and Gordon, 1974; Mishkin and Delacour, 1975; Hampson et al., 1993; Chudasama, 2010). In a typical lateralized DMTS task, participants memorize a varying number of items in a relevant hemifield; after a retaining period, the items have to be compared to a probe prompting a similarity judgment with respect to the stored mnemonic representation. The variation of memory load is instrumental to determine the individual vWM capacity limit (Cowan, 2010). The EEG marker usually extracted from this task is a posterior long-lasting deflection whose amplitude varies with increasing memory load (the so-called contralateral delay activity, CDA; for a review, see Luria et al., 2016), which has been interpreted as reflecting the number of elements maintained in the vWM buffer. The behavioral reduction in vWM capacity typical of old age is mirrored at the neural level by an attenuated CDA amplitude and/or amplitude modulation as a function of load (Jost et al., 2011; Sander et al., 2011; Störmer et al., 2013; Schwarzkopp et al., 2016; Tagliabue et al., 2019, 2020a).

This traditional, event-related potential (ERP) approach allowed researchers to disclose how older adults encode memoranda (and irrelevant items; e.g., Jost et al., 2011; Schwarzkopp et al., 2016) in the vWM buffer, and therefore why their vWM capacity is reduced in a DMTS task. However, it does not allow for the evaluation of other neurocognitive alterations that could be induced by aging. First, as the CDA is a difference waveform (computed as contralateral minus ipsilateral to targets activity), it provides no information on the separate contribution of contra- and ipsilateral channels in processing the items. Second, as the CDA is an ERP component traditionally computed over posterior electrodes, there is no information of age-related differences over other areas of the brain (e.g., over more frontal channels; but see Sander et al., 2011). With respect to the latter, it is noteworthy that sustained activity in prefrontal cortices during memory retention is a physiological correlate of WM, in both primates (Nieder, 2017) and humans (Ranganath et al., 2003). Finally, as during DMTS tasks the main focus is on the retention period (where the CDA is computed), there is scant understanding of any age-related effects in other intervals of interest, e.g., when participants compare the probe with the memory representation to produce a similarity judgment (another process that can be influenced by aging and, as a consequence, affect vWM functioning; see for example Ko et al., 2014).

Notably, according to neuroimaging studies age-related neural changes seem to follow specific patterns. Due to the consistency of the observed brain activity changes, different models of neurocognitive aging (see Festini et al., 2018 for a recent review) have been proposed to account for the neural architecture of the aging brain in several cognitive domains, including WM (e.g., Grady et al., 1994, 1995, 1999; Madden et al., 1997; Reuter-Lorenz et al., 2000; Rypma and D’Esposito, 2000; Rypma et al., 2001; Cabeza, 2002; Cappell et al., 2010). The Hemispheric Asymmetry Reduction in Older Adults (HAROLD; Cabeza, 2002) and the Posterior-Anterior Shift in Aging (PASA; Davis et al., 2008) are among the most influential models. These models are not mutually exclusive and can be considered as complementary.

According to the HAROLD theory (Cabeza, 2002), older individuals exhibit bilateral brain activations (mainly) over prefrontal areas (but see Grady et al., 2000, 2002; Nielson et al., 2002; Berlingeri et al., 2010; Learmonth et al., 2017), while their younger counterparts show a lateralized recruitment of the task-dominant hemisphere. For instance, in their seminal study Cabeza et al. (1997) found that in a word-pair learning task younger adults showed a right-lateralized activity in frontal areas during recall, whereas older adults had a more bilateral frontal activation pattern. Such reduction in asymmetry was also observed in tasks engaging other cognitive functions, e.g., working memory (Reuter-Lorenz et al., 2000), perception (Grady et al., 1994, 2000) and inhibitory control (Nielson et al., 2002).

The PASA model (Davis et al., 2008) posits that, with respect to young adulthood, the aging brain shows increased neural activity in frontal areas coupled with a reduced activation in occipitotemporal regions, as a compensatory mechanism for sensory deficits that can affect the older population (e.g., Sara and Faubert, 2000; Schneider and Pichora-Fuller, 2000; Faubert and Bellefeuille, 2002; Tagliabue et al., 2020b). The PASA pattern was also consistently found across several cognitive tasks, including attention (Cabeza et al., 2004), perception (Grady et al., 1994), working memory (Grossman et al., 2002), and episodic memory (Cabeza et al., 2004).

The functional significance of these supplementary activations, both for the HAROLD and the PASA account, is not univocal (Cabeza et al., 2018). They are often interpreted in terms of compensatory mechanisms, so that increased activation should be beneficial to behavioral performance. However, these supplementary activations are not always sufficient to achieve successful youth-like performance (attempted compensation; Cabeza and Dennis, 2013) or, in some cases, they can even be detrimental to task performance (de-differentiation; Lindenberger and Baltes, 1994; Baltes and Lindenberger, 1997).

Notably, the conclusions drawn from most of the aforementioned studies on PASA or HAROLD rely on neuroimaging data collected from fMRI and PET. Therefore, whether the supplementary activation in anterior and bilateral areas represents a stable trait of the aging brain or a dynamic event varying over time has remained unexplored. Differently from fMRI and PET, the high temporal resolution of EEG allows us to investigate neural modulations on a millisecond scale, so that the time course of age-related effects can be better defined. Indeed, a precise analysis in the time domain can provide a deeper understanding about whether the patterns of age-related activation predicted by the models are a relatively constant phenomenon, which becomes visible from the very beginning throughout all the stages of stimulus processing, or whether the additional recruitment of brain areas dynamically varies over time.

**FIGURE 1:**
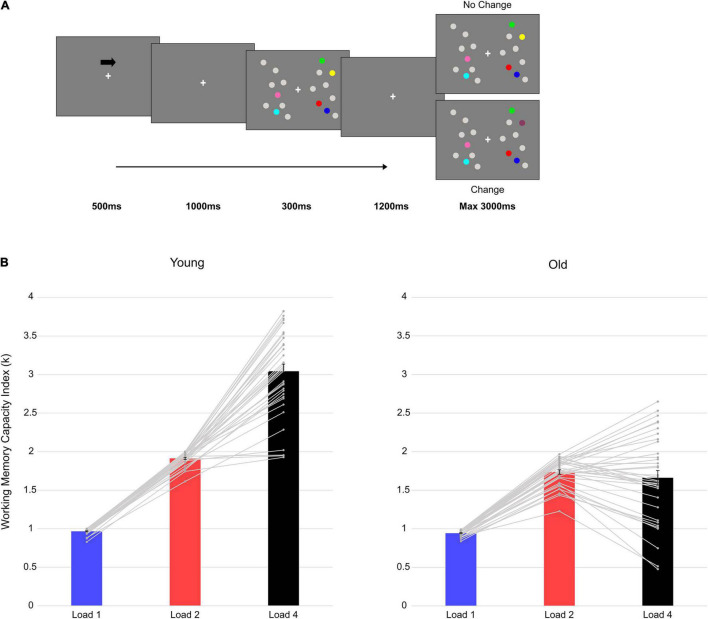
Trial structure and behavioral results. **(A)** An example with four targets (Load4) presented in the right hemifield. **(B)** Mean vWM capacity Index (k) in the two groups at each load condition. The gray lines represent single-subject data and the vertical bars represent the standard error.

Only a few EEG studies with traditional ERP analyses have so far (incidentally) investigated these accounts of neurocognitive aging (e.g., Daffner et al., 2011; Kropotov et al., 2016; Learmonth et al., 2017). The results indicated that ERP modulations in aging mirrored PASA-like activations (i.e., smaller component amplitude in posterior areas and larger over prefrontal sites) in a go/no-go task (cognitive control; Kropotov et al., 2016) and were consistent with HAROLD (i.e., no lateralized ERP responses) in a landmark task (spatial attention; Learmonth et al., 2017). Moreover, with specific reference to the WM domain, evidence of both age-related increased frontal activity (i.e., larger anterior component) and reduced asymmetry (i.e., equal component amplitude in one hemisphere, increased in the other) were found in a verbal n-back paradigm (Daffner et al., 2011). However, due to the nature of this ERP analysis approach, previous studies could not provide a precise clue about the temporal dynamics of these age-related neural phenomena. Thus, several aspects have remained unexplored. For instance, do the reduction in asymmetry and the posterior-to-anterior shift occur at all stages of stimulus processing? Once activated, do they persist across subsequent processing stages? Do HAROLD- and PASA-like activations independently evolve over time? To answer these questions, we exploited the well-defined temporal structure of the DMTS paradigm (see Figure 1A), where the different stages of stimulus processing can be easily segregated. In addition, we performed a data-driven cluster-based analysis (Oostenveld et al., 2011) over all electrodes and time points. Without an *a priori* selection of regions (ROIs) or time points (TOIs) of interest, as usually done in more traditional ERP analyses, this approach allowed us to evaluate contra- and ipsilateral activity over anterior and posterior electrode sites when processing different memory loads and, crucially, to observe when HAROLD- and PASA-like neural modulations occur (i.e., if they are present in a specific processing stage) and how they vary over time (i.e., if they also change within a specific processing stage). Indeed, the cluster-based analysis was carried out both during memory retention (as traditionally done by other vWM studies) and in the retrieval phase of item comparison.

Specifically, the separate evaluation of contra- and ipsilateral amplitude modulations as a function of load gave us the opportunity to investigate HAROLD-like activation patterns. Indeed, hemispheric (a)symmetry can be conceived not only in absolute terms (i.e., left or right hemisphere, or dominant/non-dominant hemisphere for a specific task, as previously done in fMRI and PET studies, e.g., Cabeza, 2002), but also with respect to the hemisphere that is contralateral or ipsilateral relative to the hemifield of the relevant items, in the case of lateralized presentations. In line with age-related reduced hemispheric asymmetry, older adults should exhibit amplitude modulations as a function of memory load in both contra- and ipsilateral channels, when the same modulation is mainly visible in contralateral electrodes in the younger group. As for the evaluation of PASA-related dynamics, we expected load-related modulations not only in posterior channels for the older adults, but also over more anterior sites (and larger than in young adulthood).

## Materials and Methods

Part of the data from two previous studies were used (Tagliabue et al., 2019, 2020a). In both studies, we used exactly the same experimental paradigm and parameters. In these two studies, we were interested in how vWM capacity was influenced by numerical similarity (Tagliabue et al., 2019) and repetition-learning (Tagliabue et al., 2020a) in older and younger adults, and how these modulations were specifically reflected in changes in the posterior CDA component during memory retention (i.e., after the memory array presentation, see Figure 1A). Here we analyzed the data (only the first session of Tagliabue et al., 2020a) adopting a new, data-driven whole-brain approach, and for both item retention (i.e., after memory array presentation, see Figure 1A), and item comparison (i.e., during the test array, see Figure 1A) stages.

### Participants

Participants’ data with at least 65% of their EEG trials retained after artifact rejection were included, thus resulting in a final sample of 36 healthy young adults (18 males; mean age 23.83 ± 3.39; mean years of school 15.61 ± 1.83) and 35 healthy old adults (15 males; mean age 69.89 ± 4.86; mean years of school 12.34 years ± 2.04). To exclude any suspect of cognitive impairment, participants from the older group underwent a neuropsychological assessment that comprised also the Mini Mental State Examination (MMSE; Measso et al., 1993; average correct score 28.33 ± 1.60; reference pathological cut-off score ≤ 23.80).

### Stimuli and Procedure

The two groups performed a lateralized DMTS task. For specific details, the reader is referred to Tagliabue et al. (2019). In sum, an arrow cue (500 ms, 100% valid) indicated the relevant hemifield (left/right) for which participants had to memorize the colored dots presented. After cue disappearance and a 1,000-ms delay, a memory array of gray and colored dots was presented for 300 ms in both hemifields (a total of nine gray and colored dots in each hemifield). Participants’ task was to remember the color of the colored dots in the relevant (i.e., cued) hemifield. Either one, two, or four colored dots, together with gray dots, were independently presented in the two hemifields. After a retention interval of 1,200 ms, a test array was presented for a maximum time of 3,000 ms. Participants were asked to report whether the test array was different (i.e., change condition: one of the colored targets in the relevant hemifield changed color; 50% of the trials) or not (i.e., no-change condition: the colored targets in the relevant hemifield did not change color; 50% of the trials) compared to the memory array (Figure 1A), by pressing the respective key button on the keyboard (letter M or C, counterbalanced across participants). Participants performed a total of 720 trials (240 trials for each memory load—one, two, four) divided in 15 blocks of 48 trials each.

### Electroencephalography Recording and Analysis

The EEG signal was continuously recorded during the lateralized DMTS task through 29 active electrodes, with a sampling rate of 1,000 Hz, a time constant of 10 s as low cut-off and 250 Hz of high-cutoff filters. The right mastoid served as on-line reference, and AFz as ground electrode. Offline pre-processing analysis was performed on the continuous EEG recording using EEGLAB (Delorme and Makeig, 2004) and ERPLAB (Lopez-Calderon and Luck, 2014).

Raw EEG data were down sampled to 250 Hz and filtered with high- and low cut-off filters of 40 and 0.1 Hz, respectively. Moreover, a 50 Hz notch filter (width: 2 Hz) was applied. The activity of all channels was re-referenced to the average of left and right mastoids. The continuous signal was then segmented in 5-s long epochs, starting -2,500 ms before to 2,500 ms after memory array onset. Independent Component Analysis (ICA) was applied to the segmented dataset (Groppe et al., 2009) in order to identify artifactual activity (e.g., eye blinks, saccadic eye movements, muscle and cardiac activity; Liesefeld et al., 2014). The artifactual components were manually removed after visual inspection of their topography and activity over time. Then, epochs were baseline corrected over the whole epoch length and residual noise was removed by eliminating epochs in which activity exceeded ± 150 μV in one or more channels.

Only epochs yielding a correct response were selected for further analyses. For EEG responses to both memory and test array, three datasets (one for each load condition, i.e., Load1, Load2 and Load4) were created. Epochs were collapsed across change condition (change/no-change in the test array) and target side (left/right), to obtain activity contralateral and ipsilateral to the target location, regardless of the actual direction of the cue and side of presentation of the target (i.e., if targets are presented in the left hemifield, right electrodes are contralateral while left electrodes are ipsilateral, and vice-versa for right targets). For the analysis of the time window following the memory array, a -200 to 0 ms baseline correction was applied (i.e., 200 ms preceding memory array onset). For the analysis of the EEG activity in response to the test array, trials were baseline corrected over the 200 ms preceding test array onset. The mean number of epochs retained was: 226.08 (*SD* = 13.92) for Load1, 223.75 (*SD* = 15.72) for Load2 and 199.36 (*SD* = 20.14) for Load4 in younger adults; 223.37 (*SD* = 10.69) for Load1, 214.29 (*SD* = 13.99) for Load2 and 161.77 (*SD* = 16.92) for Load4 in older adults.

### Statistical Analysis

#### Behavioral Data

The vWM capacity index (i.e., k) was calculated using the following formula: *k* = [(hit rate - false alarm rate)/(1 - false alarm rate)] * load, where load refers to the number of colored dots presented in the target hemifield (Pashler, 1988; Rouder et al., 2011). Hits represent correct “different” responses during change conditions, while false alarms incorrect “different” responses in no-change trials (Vogel and Machizawa, 2004; Vogel et al., 2005). The *k*-value indicates the number of items retained in the memory buffer and increases with increasing memory load until reaching a plateau at the individual vWM limit: higher *k*-values are considered as a proxy of higher vWM capacity.

A mixed analysis of variance (ANOVA) was conducted on k measures with Group as a between-subjects factor (two levels: Old, Young), and Load as a within-subjects variable (three levels: Load1, 2 and 4).

#### Electroencephalography Data

The analysis of EEG data was conducted in two steps.

First, a non-parametric cluster-based permutation analysis was performed as implemented in Fieldtrip (Oostenveld et al., 2011). This data-driven approach allows us to observe the spatio-temporal pattern of the effects of interest, which is most informative when dealing with different age groups. Indeed, ERPs might have different topographical distributions (Mueller et al., 2008) or latencies (Gazzaley et al., 2005) when dealing with different age cohorts. In order to investigate the main effect of **Load**, within each age group a non-parametric cluster-based permutation ANOVA was performed at each channel × time point sample, from 0 to 1,200 ms after memory array onset (item retention) and from 0 to 500 ms after test array onset (item comparison, i.e., from 1,500 to 2,000 ms after memory array onset, see Figure 1A; the latter time interval was determined to avoid potential confounds due to manual response). To investigate the presence of a **Load × Group** interaction, as suggested in the Fieldtrip documentation (Oostenveld et al., 2011) we performed at each channel × time point pair an independent-samples *t*-test on the Load effect (i.e., Load1—Load4 difference computed for each group), in the same time windows used for the ANOVAs. For all the analyses, the channel × time point pairs where the *F* or *t-*value exceeded a critical value (*p* < 0.05) were selectively clustered on the basis of spatial and temporal adjacency. Neighboring channels were selected based on a triangulation method. Within each selected cluster, *F/t*-values were summed to calculate cluster-level statistics later evaluated through a non-parametric permutation test with 1,000 iterations. At each permutation, cluster-based statistics were computed and a reference distribution was created. The proportion of random partitions exceeding the maximum cluster statistic finally provides the Monte Carlo significance *p*-value (critical alpha value for *F*-tests: 0.05; for *t*-tests: 0.025). This analysis allowed us to test, without any *a priori* selection of electrodes and time points, HAROLD- and PASA-like modulations. The presence of a significant cluster for the Load effect over bilateral electrodes in older adults would be in line with HAROLD assumptions (i.e., reduced asymmetry), while a significant cluster for the Load effect over anterior channels would resemble PASA activations (i.e., shift toward anterior areas).

As a second step, we ran traditional analyses by computing the mean amplitude over ROIs and TOIs based on previous literature (Hillyard et al., 1998; Spencer and Polich, 1999; Vogel and Machizawa, 2004; Sander et al., 2011; Störmer et al., 2013; Feldmann-Wüstefeld et al., 2018). These additional analyses were performed to ensure convergence of results between the cluster-based permutation and the traditional ERP approach. Following memory array presentation, we computed the mean amplitude in a 500-ms time window (from 300 to 800 ms after memory array onset; see Feldmann-Wüstefeld et al., 2018) over posterior contra- and ipsilateral electrodes PO7/8, P7/8 and P3/4, where the CDA (Vogel and Machizawa, 2004) is usually observed, and over anterior contra- and ipsilateral electrodes Fp1/2, F3/4, and F7/8 (see Sander et al., 2011). As there are no aging studies in literature investigating the probe array phase with such experimental structure, we decided to compute the mean amplitude in time windows corresponding to components usually elicited by targets in visual tasks, i.e., the P1 (80–100 ms after probe onset, as a marker of perceptual processing; Hillyard et al., 1998), the N1 (140–190 ms after probe onset, as a marker of early attentional processing; Hillyard et al., 1998) and the P300 (250–400 ms after probe onset, as a marker of stimulus categorization; Spencer and Polich, 1999), over the same anterior/posterior and contra/ipsilateral channels used for the memory array analysis.

Separately for each event (memory and probe array) and for each component (P1, N1, and P300 only for the probe array), we ran mixed ANOVAs with Group as between-subjects factor and Load (3 levels: Load 1, 2, and 4), ROI (2 levels: posterior, anterior) and hemisphere (2 levels: contralateral, ipsilateral) as within-subjects factors. For all the components, we were interested in load-related modulation differences between the two groups, so that only significant interactions involving the factors Group and Load will be discussed. Indeed, the presence of a significant load effect is a proxy of the neural efficiency in discriminating to-be-memorized items (see also Tagliabue et al., 2020a). Similar to the non-parametric cluster-based permutation analysis, we looked for the presence of Load-related modulations in a more traditional ERP approach by selecting well-known ERP components across different ROIs. A significant Load effect over bilateral ROIs in older adults (in one or more ERP components) would suggest HAROLD-like modifications, while the presence of Load-related modulations over frontal ROIs is in line with the PASA account.

For both behavioral data and ERP components, interactions and main effects were investigated through subsequent ANOVAs and/or *post hoc* analyses by means of polynomial contrasts (adjusted for unequal spacing between load levels, i.e., 1, 2, 4). In case of violation of sphericity Greenhouse–Geisser (when G–G epsilon < 0.75) or Huynh–Feldt (when G–G epsilon > 0.75) correction was used and adjusted *p*-values are reported.

## Results

### Behavioral Data

Descriptive statistics of the behavioral variables (*k*-values) are reported in Supplementary Table 1.

The ANOVA on the vWM capacity index (k) showed significant main effects of Group [*F*_(1, 69)_ = 97.462, *p* < 0.001, η*_*p*_*^2^ = 0.585], Load [*F*_(2, 138)_ = 403.142, *p* < 0.001, η*_*p*_*^2^ = 0.854], and a significant interaction between the two factors [*F*_(2, 138)_ = 112.184, *p* < 0.001, η*_*p*_*^2^ = 0.619].

In **younger individuals**, a significant main effect of Load emerged [*F*_(2, 70)_ = 425.081, *p* < 0.001, η*_*p*_*^2^ = 0.924]. *Post hoc* polynomial contrast tests showed a significant quadratic trend [*F*_(1, 35)_ = 86.433, *p* < 0.001, η*_*p*_*^2^ = 0.712], suggesting that vWM capacity increased until reaching a limit of around three elements retained (mean k at Load4 = 3.04). Also in **older adults** Load was significant [*F*_(2, 68)_ = 80.202, *p* < 0.001, η*_*p*_*^2^ = 0.702] and *post hoc* polynomial contrasts revealed a significant quadratic trend [*F*_(1, 34)_ = 766.989, *p* < 0.001, η*_*p*_*^2^ = 0.958]. The increase in vWM capacity with load reached a plateau at less than two elements (mean k at Load2 = 1.74). Follow-up analyses (independent-samples *t*-tests; Bonferroni corrected alpha level: 0.05/2 = 0.025) compared the performance of the two groups at the two extreme loads (i.e., Load1 and 4). Even if with a slight difference at Load1 (0.94 vs. 0.97 for older and younger, respectively), younger adults outperformed older participants at both loads [Load1: *t*(69) = 2.580, *p* = 0.012, *d* = 0.612, 95% CI = (−0.041 −0.05); Load4: *t*(69) = 10.490, *p* < 0.001, *d* = 2.490, 95% CI = (−1.646 −1.120)]. Finally, the increase in vWM capacity (Load4—Load1 difference) was compared between the two groups. The test was significant [*t*(69) = −10.700, *p* < 0.001, *d* = 2.540, 95% CI = (−1.613 −1.106)], indicating a larger increase in the younger group.

Overall, younger outperformed older participants. Indeed, older adults reached a plateau in their vWM capacity earlier than younger individuals (Figure 1B).

### Electroencephalography Data

#### Item Retention Phase

##### Non-parametric Cluster-Based Permutation Analysis

**FIGURE 2:**
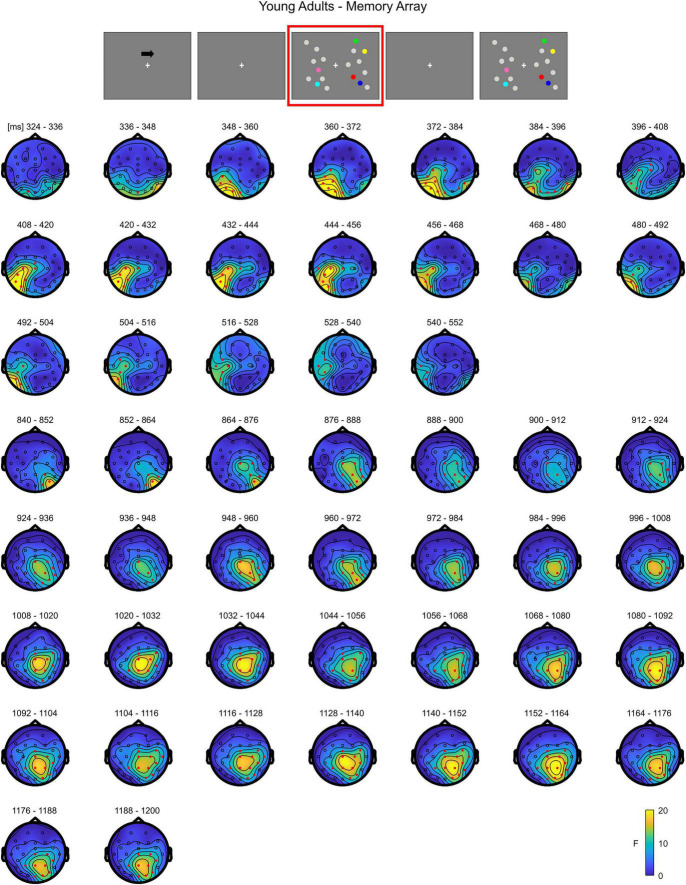
Cluster-based permutation ANOVA results during memory retention in the Younger group. The spatio-temporal evolution of the significant Load effect is represented through topographical maps of the clusters averaged in bins of 12 ms. Only significant time windows are depicted (for the complete time course, see Supplementary Figure 1). Red dots represent the significant effect. The color bar represents the range of *F*-values. Left and right channels are set as contralateral and ipsilateral to targets, respectively.

In **younger adults**, the cluster-based permutation ANOVA in the latency range from 0 to 1,200 ms after memory array onset revealed a significant Load effect approximately between 320 and 556 ms (positive cluster, *p* = 0.023) and 836–1,200 ms (positive cluster, *p* = 0.014) post stimulus onset. In the first time window (320–556 ms), the cluster of the effect was evident over posterior contralateral channels. In the second time window (836–1,200 ms), the effect was initially pronounced over posterior channels ipsilateral to target side and later evolving (around 990 ms) to a more central topography (Figure 2; as target side was collapsed, left and right channels are set as contralateral and ipsilateral to targets, respectively. For a visualization of the entire time course of the effect, see Supplementary Figure 1).

**FIGURE 3:**
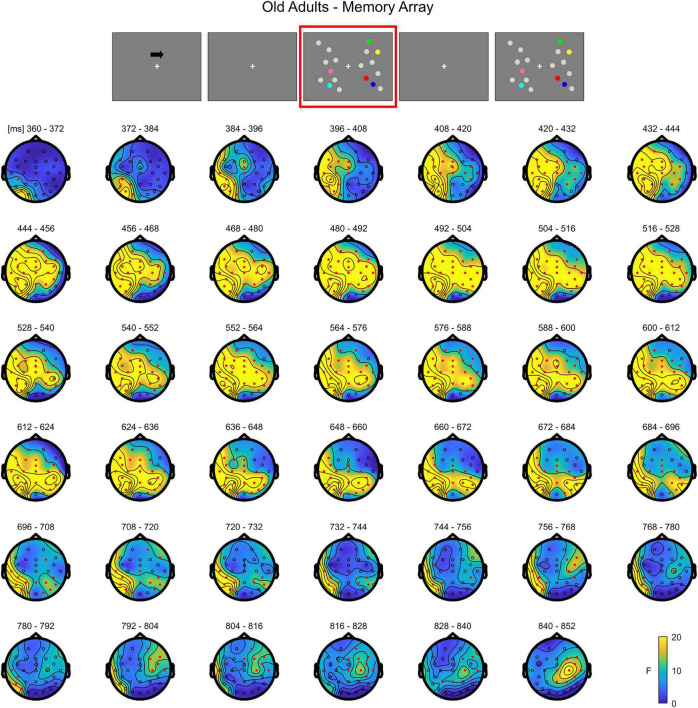
Cluster-based permutation ANOVA results during memory retention in the Older group. The spatio-temporal evolution of the significant Load effect is represented through topographical maps of the clusters averaged in bins of 12 ms. Only significant time windows are depicted (for the complete time course, see Supplementary Figure 2). Red dots represent the significant effect. The color bar represents the range of *F*-values. Left and right channels are set as contralateral and ipsilateral to targets, respectively.

In **older adults**, the cluster-based permutation ANOVA showed a significant effect between 356 and 876 ms (positive cluster, *p* = 0.001). By observing the spatio-temporal evolution of the effect, the load-related modulation was initially pronounced (360–390 ms) over posterior contralateral electrodes, but soon evolved to comprise both frontal channels and the ipsilateral hemisphere, finally restraining to a more central-anterior ipsilateral topography (around 790 ms) (Figure 3; see Supplementary Figure 2 for the whole time course of the effect).

**FIGURE 4:**
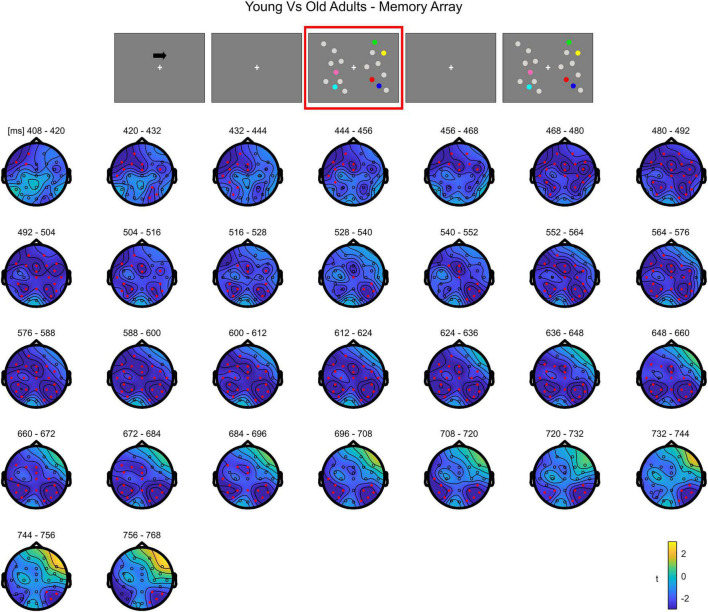
Cluster-based permutation independent-samples *t*-test results on the load effect between the two groups during memory retention. The spatio-temporal evolution of the significant Group effect is represented through topographical maps of the clusters averaged in bins of 12 ms. Only significant time windows are depicted (for the complete time course, see Supplementary Figure 3). Red dots represent the significant effect. The color bar represents the range of *t*-values. Left and right channels are set as contralateral and ipsilateral to targets, respectively.

The cluster-based permutation independent *t*-test on the load effect (Load1–Load4 difference) between the two groups indicated a significant difference spanning from 400 to 768 ms (negative cluster, *p* = 0.003). Older participants exhibited a larger load effect than younger adults. The difference was initially more pronounced over contralateral channels (both posterior and anterior) and later (from around 470 ms) on the whole scalp (Figure 4; see Supplementary Figure 3 for the complete time course).

Overall, the results show that in younger adults the processing of memory loads is mostly evident over posterior electrodes. Notably, this effect is observed contralaterally to targets right after the presentation of the memory array, evolving to a more central topography toward the end of the retention period. Conversely, in the older group, memory load processing quickly evolves toward frontal channels, and broadly spreads bilaterally.

##### Mean Amplitude Analysis

Descriptive statistics of the EEG variables during item retention are reported in Supplementary Table 2.

The ANOVA revealed significant main effects of Group [*F*_(1, 69)_ = 28.512, *p* < 0.001, η*_*p*_*^2^ = 0.292], Load [*F*_(2, 138)_ = 7.975, *p* < 0.001, η*_*p*_*^2^ = 0.104], ROI [*F*_(1, 69)_ = 4.410, *p* = 0.039, η*_*p*_*^2^ = 0.060] and Hemisphere [*F*_(1, 69)_ = 71.810, *p* < 0.001, η*_*p*_*^2^ = 0.510]. The following interactions were also significant: Load × Group [*F*_(2, 138)_ = 6.094, *p* = 0.003, η*_*p*_*^2^ = 0.081], ROI × Group [*F*_(1, 69)_ = 4.410, *p* = 0.002, η*_*p*_*^2^ = 0.125], Hemisphere × Group [*F*_(1, 69)_ = 17.518, *p* < 0.001, η*_*p*_*^2^ = 0.202], Load × Hemisphere [*F*_(2, 138)_ = 38.542, *p* < 0.001, η*_*p*_*^2^ = 0.358], Load × ROI × Hemisphere [*F*_(2, 138)_ = 13.270, *p* < 0.001, η*_*p*_*^2^ = 0.161] and, finally, the four-way interaction **Load × ROI × Hemisphere × Group** [*F*_(2, 138)_ = 6.803, *p* = 0.002, η*_*p*_*^2^ = 0.090].

**FIGURE 5:**
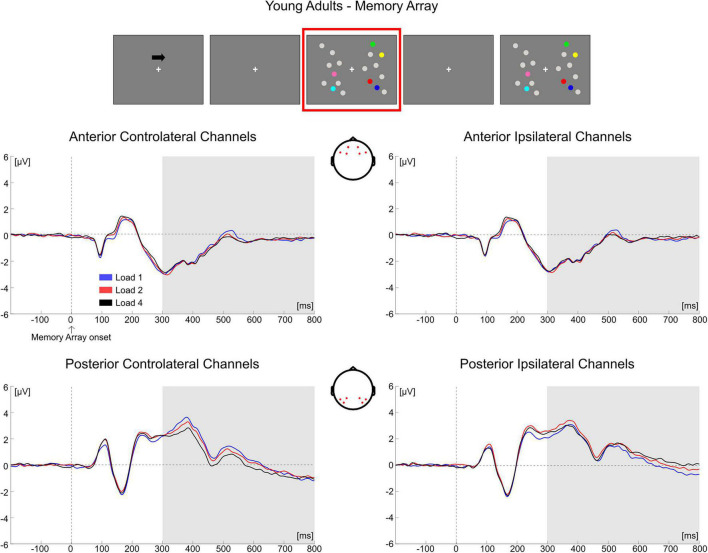
Grand average waveforms as a function of target load over the anterior (Fp1/2, F3/4, F7/8) and posterior ROI (P7/8, PO7/8, P3/4), separately for contra- and ipsilateral to target channels, in the Younger group during memory retention. Topographies highlight the channels included in the ROIs and gray squares indicate the TOIs over which the mean amplitude was computed.

In **younger adults**, the follow-up ANOVA indicated significant main effects of ROI [*F*_(1, 35)_ = 14.824, *p* < 0.001, η*_*p*_*^2^ = 0.298] and Hemisphere [*F*_(1, 35)_ = 20.728, *p* < 0.001, η*_*p*_*^2^ = 0.372] and significant interactions for Load × Hemisphere [*F*_(2, 70)_ = 12.123, *p* < 0.001, η*_*p*_*^2^ = 0.257], ROI × Hemisphere [*F*_(1, 35)_ = 9.117, *p* = 0.005, η*_*p*_*^2^ = 0.207] and Load × ROI × Hemisphere [*F*_(2, 70)_ = 20.083, *p* < 0.001, η*_*p*_*^2^ = 0.365]. In posterior channels, a significant main effect of Hemisphere [*F*_(1, 35)_ = 21.201, *p* < 0.001, η*_*p*_*^2^ = 0.377] and a significant Load × Hemisphere interaction [*F*_(2, 70)_ = 23.337, *p* < 0.001, η*_*p*_*^2^ = 0.400] were found, with only contralateral channels exhibiting a Load effect [*F*_(2, 70)_ = 3.367, *p* = 0.048, η*_*p*_*^2^ = 0.088] reflecting a linear decrease in amplitude with increasing memory load [linear trend: *F*_(1, 35)_ = 4.953, *p* = 0.033, η*_*p*_*^2^ = 0.124]. There was no significant effect of load in posterior ipsilateral channels (*p* = 0.104). In anterior channels, there were no significant effects (all *p*s > 0.140) (Figure 5).

**FIGURE 6:**
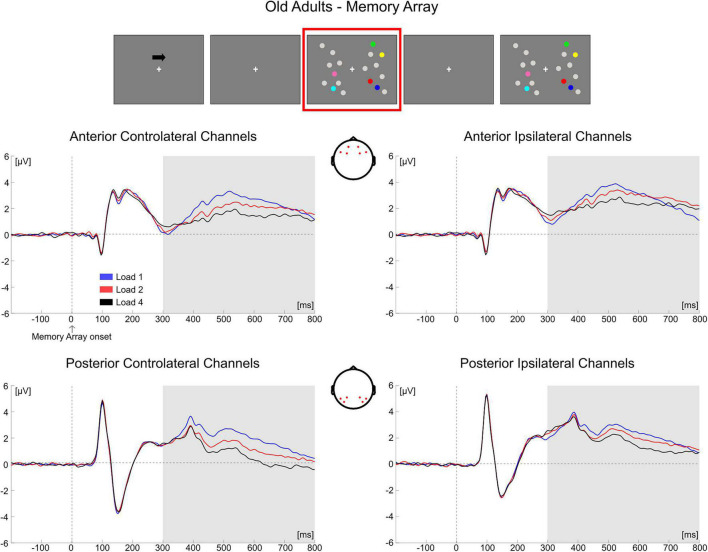
Grand average waveforms as a function of target load over the anterior (Fp1/2, F3/4, F7/8) and posterior ROI (P7/8, PO7/8, P3/4), separately for contra- and ipsilateral to target channels, in the Older group during memory retention. Topographies highlight the channels included in the ROIs and gray squares indicate the TOIs over which the mean amplitude was computed.

In **older adults**, the follow-up ANOVA showed main effects of Load [*F*_(2, 68)_ = 13.281, *p* < 0.001, η*_*p*_*^2^ = 0.281] and Hemisphere [*F*_(1, 34)_ = 50.454, *p* < 0.001, η*_*p*_*^2^ = 0.597] and a significant Load × Hemisphere interaction [*F*_(2, 68)_ = 31.366, *p* < 0.001, η*_*p*_*^2^ = 0.480]. A significant load effect was evident in both the contralateral [*F*_(2, 68)_ = 24.964, *p* < 0.001, η*_*p*_*^2^ = 0.423] and ipsilateral hemisphere [*F*_(2, 68)_ = 5.058, *p* = 0.014, η*_*p*_*^2^ = 0.130], indicating for all channels a linear decrease in amplitude as memory load increased [linear trends: contralateral, *F*_(1, 34)_ = 34.688, *p* < 0.001, η*_*p*_*^2^ = 0.505; ipsilateral, *F*_(1, 34)_ = 6.524, *p* = 0.015, η*_*p*_*^2^ = 0.161] (Figure 6).

Conditioned on the results obtained in the two groups over the posterior contralateral area (the only ROI where a load-related modulation was found in the younger group), we performed a follow-up analysis. We compared the load-related modulation (Load1—Load4 difference) in posterior contralateral channels between the two groups. The test was significant [*t*(69) = −2.766, *p* = 0.007, *d* = 0.657, 95% CI = (−1.198 −0.194)], showing a larger load effect in older participants.

Overall, the results obtained from the mean amplitude ERP analysis were in line with what was observed through the cluster-based permutation tests. In the younger group, the posterior electrodes contralateral to the targets were mainly modulated as a function of memory load. Older adults exhibited instead a load-related modulation both in contralateral and ipsilateral channels; the modulation encompassed both posterior and anterior ROIs.

#### Item Comparison Phase

##### Non-parametric Cluster-Based Permutation Analysis

**FIGURE 7:**
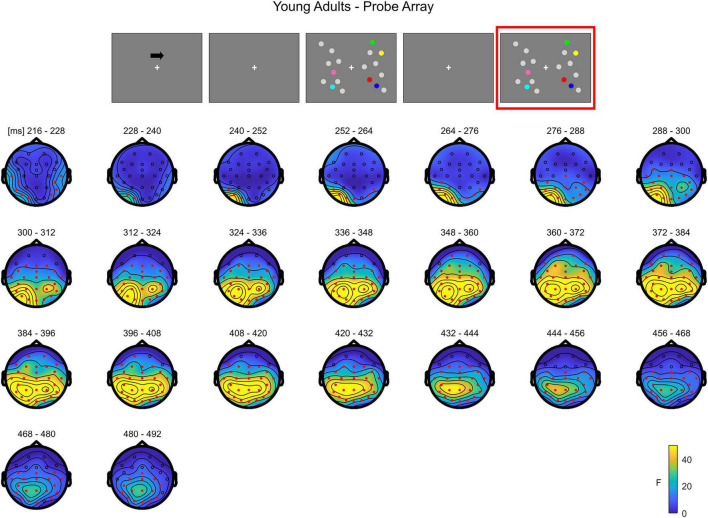
Cluster-based permutation ANOVA results during item comparison in the Younger group. The spatio-temporal evolution of the significant Load effect is represented through topographical maps of the clusters averaged in bins of 12 ms. Only significant time windows are depicted (for the complete time course, see Supplementary Figure 4). Red dots represent the significant effect. The color bar represents the range of *F*-values. Left and right channels are set as contralateral and ipsilateral to targets, respectively.

In **younger adults**, the cluster-based permutation ANOVA in the latency range from 0 to 500 ms following test array onset showed a significant Load effect between 208 and 500 ms (positive cluster, *p* = 0.001). In this time window, the effect was initially (∼208–290 ms) more evident over posterior channels bilaterally, later (from approximately 300 ms post-stimulus onset) spreading also to anterior sites (Figure 7; see Supplementary Figure 4 for the whole time course).

**FIGURE 8:**
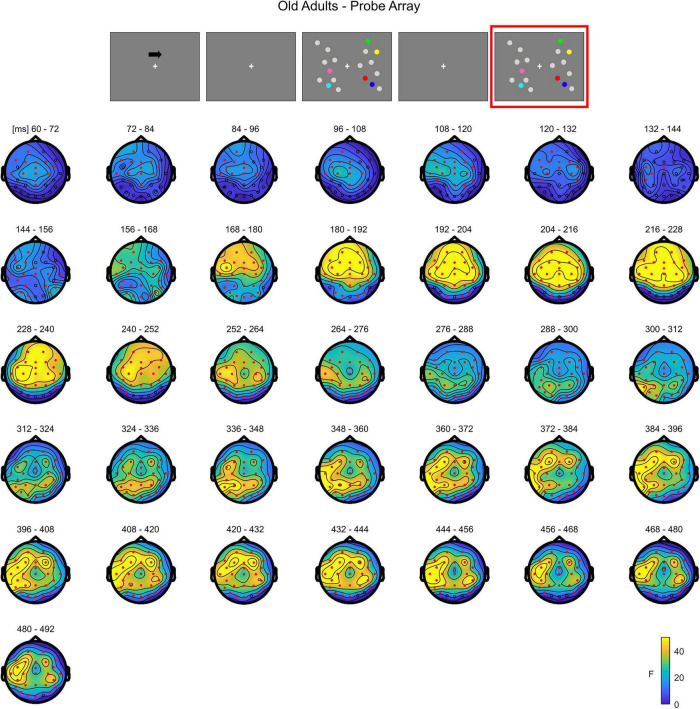
Cluster-based permutation ANOVA results during item comparison in the Older group. The spatio-temporal evolution of the significant Load effect is represented through topographical maps of the clusters averaged in bins of 12 ms. Only significant time windows are depicted (for the complete time course, see Supplementary Figure 5). Red dots represent the significant effect. The color bar represents the range of *F*-values. Left and right channels are set as contralateral and ipsilateral to targets, respectively.

The non-parametric test in the **older group** revealed a significant effect between 52 and 500 ms (positive cluster, *p* = 0.001). The effect was initially more pronounced over anterior areas (∼52–140 ms), and later evolved (around 140 ms) to a more widespread topography (Figure 8; see Supplementary Figure 5 for the complete time course).

**FIGURE 9:**
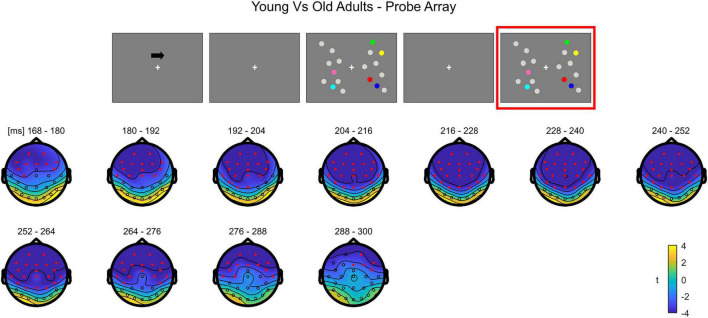
Cluster-based permutation independent-samples *t*-test results on the load effect between the two groups during item comparison. The spatio-temporal evolution of the significant Group effect is represented through topographical maps of the clusters averaged in bins of 12 ms. Only significant time windows are depicted (for the complete time course, see Supplementary Figure 6). Red dots represent the significant effect. The color bar represents the range of *t*-values. Left and right channels are set as contralateral and ipsilateral to targets, respectively.

The cluster-based permutation test on the load effect (Load1—Load4) between the two groups indicated a difference between younger and older individuals from 160 to 308 ms (negative cluster, *p* = 0.001). In this latency, older adults exhibited a larger load effect initially over anterior electrodes, later (around 460 ms) encompassing almost the whole scalp, with the exception of the most posterior channels (Figure 9; see Supplementary Figure 6 for the whole time course).

To summarize, during the test array younger individuals exhibited a load-related modulation at later latencies. Although the effect had initially a more posterior topography, it was then strongly evident over the whole scalp. In the older group, the effect was evident earlier in time over anterior sites, then evolving to a more widespread topographical distribution.

##### Mean Amplitude Analyses

Descriptive statistics of the EEG variables during item comparison are reported in Supplementary Table 3.

*80–100 ms*. The mixed ANOVA returned significant main effects of Group [*F*_(1, 69)_ = 18.222, *p* < 0.001, η*_*p*_*^2^ = 0.209], Load [*F*_(2, 138)_ = 4.055, *p* = 0.019, η*_*p*_*^2^ = 0.056) and ROI [*F*_(1, 69)_ = 101.242, *p* < 0.001, η*_*p*_*^2^ = 0.595] and the following significant interactions: ROI × Group [*F*_(1, 69)_ = 14.779, *p* < 0.001, η*_*p*_*^2^ = 0.176], Hemisphere × Group [*F*_(1, 69)_ = 17.019, *p* < 0.001, η*_*p*_*^2^ = 0.198], **Load × ROI × Group** [*F*_(2, 138)_ = 11.633, *p* < 0.001, η*_*p*_^2^* = 0.144) and ROI × Hemisphere × Group [*F*_(1, 69)_ = 19.510, *p* < 0.001, η_*p*_^2^ = 0.220].

We investigated only the Load × ROI × Group interaction as it included the load factor (see “Electroencephalography Data” section). The subsequent ANOVA in **younger adults** revealed only a significant main effect of ROI [*F*_(1, 35)_ = 45.654, *p* < 0.001, η*_*p*_*^2^ = 0.566], with posterior electrodes exhibiting larger positive amplitude than anterior ones.

In the **older group**, significant effects for Load [*F*_(2, 68)_ = 3.566, *p* = 0.034, η*_*p*_*^2^ = 0.095], ROI [*F*_(1, 34)_ = 60.068, *p* < 0.001, η*_*p*_*^2^ = 0.639] and Load × ROI [*F*_(2, 68)_ = 11.606, *p* < 0.001, η*_*p*_*^2^ = 0.254] emerged. While in posterior electrodes no load-related modulation was evident (*p* = 0.557), it was instead significant over anterior sites [*F*_(2, 68)_ = 8.915, *p* < 0.001, η*_*p*_*^2^ = 0.208], with the amplitude of the component becoming more negative as load increased [linear trend: *F*_(1, 34)_ = 14.711, *p* = 0.001, η_*p*_^2^ = 0.302].

*140–190 ms*. The main ANOVA found significant effects for Load [*F*_(2, 138)_ = 9.130, *p* < 0.001, η*_*p*_*^2^ = 0.117], ROI [*F*_(1, 69)_ = 84.089, *p* < 0.001, η*_*p*_*^2^ = 0.549], Hemisphere × Group [*F*_(1, 69)_ = 20.204, *p* < 0.001, η*_*p*_*^2^ = 0.226], Load × ROI [*F*_(2, 138)_ = 22.976, *p* < 0.001, η*_*p*_*^2^ = 0.250], Load × Hemisphere [*F*_(2, 138)_ = 6.003, *p* = 0.005, η*_*p*_*^2^ = 0.080], ROI × Hemisphere [*F*_(1, 69)_ = 4.153, *p* = 0.045, η*_*p*_*^2^ = 0.057], **Load × ROI × Group** [*F*_(2, 138)_ = 15.304, *p* < 0.001, η*_*p*_*^2^ = 0.182], ROI × Hemisphere × Group [*F*_(1, 69)_ = 16.966, *p* < 0.001, η*_*p*_*^2^ = 0.197] and Load × ROI × Hemisphere [*F*_(2, 138)_ = 15.910, *p* < 0.001, η*_*p*_*^2^ = 0.187].

Again, we decomposed only the Load × ROI × Group interaction. In the **younger group**, the subsequent ANOVA to decompose the three-way Load × ROI × Group interaction found only a significant main effect of ROI [*F*_(1, 35)_ = 18.475, *p* < 0.001, η*_*p*_*^2^ = 0.345], showing that posterior electrodes had more negative amplitude than anterior channels.

From the ANOVA in the **older group** all the effects were significant: Load [*F*_(2, 68)_ = 8.502, *p* < 0.001, η*_*p*_*^2^ = 0.200], ROI [*F*_(1, 34)_ = 111.203, *p* < 0.001, η_*p*_^2^ = 0.766] and Load × ROI [*F*_(2, 68)_ = 30.234, *p* < 0.001, η*_*p*_*^2^ = 0.471]. In both posterior [*F*_(2, 68)_ = 7.554, *p* = 0.001, η_*p*_^2^ = 0.182] and anterior electrodes [*F*_(2, 68)_ = 20.090, *p* < 0.001, η*_*p*_*^2^ = 0.371] there was a significant load effect. *Post hoc* polynomial contrasts showed a significant quadratic trend in both ROIs (posterior: quadratic [*F*_(1, 34)_ = 8.001, *p* = 0.008, η*_*p*_*^2^ = 0.190]; anterior: quadratic [*F*_(1, 34)_ = 14.218, *p* = 0.001, η*_*p*_*^2^ = 0.295]), with amplitudes becoming less negative with load in posterior electrodes and the reverse occurring in the anterior ROI.

*250–400 ms*. The mixed ANOVA showed significant main effects for Load [*F*_(2, 138)_ = 60.048, *p* < 0.001, η*_*p*_*^2^ = 0.465], ROI [*F*_(1, 69)_ = 20.750, *p* < 0.001, η*_*p*_*^2^ = 0.231] and Hemisphere [*F*_(1, 69)_ = 22.118, *p* < 0.001, η*_*p*_*^2^ = 0.243] and the following significant interactions: ROI × Group [*F*_(1, 69)_ = 14.142, *p* < 0.001, η*_*p*_*^2^ = 0.170], Hemisphere × Group [*F*_(1, 69)_ = 5.779, *p* = 0.019, η*_*p*_*^2^ = 0.077], Load × ROI [*F*_(2, 138)_ = 5.495, *p* = 0.008, η*_*p*_*^2^ = 0.074], Load × Hemisphere [*F*_(2, 138)_ = 15.105, *p* < 0.001, η*_*p*_*^2^ = 0.180], ROI × Hemisphere [*F*_(1, 69)_ = 9.648, *p* = 0.003, η*_*p*_*^2^ = 0.123], **Load** × **ROI** × **Group** [*F*_(2, 138)_ = 13.181, *p* < 0.001, η*_*p*_*^2^ = 0.160] and Load × ROI × Hemisphere [*F*_(2, 138)_ = 8.397, *p* < 0.001, η*_*p*_*^2^ = 0.108].

In the **younger group**, the following ANOVA to investigate the Load × ROI × Group interaction found significant effects of Load [*F*_(2, 70)_ = 27.664, *p* < 0.001, η*_*p*_*^2^ = 0.441], ROI [*F*_(1, 35)_ = 30.022, *p* < 0.001, η*_*p*_*^2^ = 0.462] and Load × ROI [*F*_(2, 70)_ = 22.361, *p* < 0.001, η*_*p*_*^2^ = 0.390]. There was a significant load effect in both posterior [*F*_(2, 70)_ = 48.653, *p* < 0.001, η*_*p*_*^2^ = 0.582] and anterior channels [*F*_(2, 70)_ = 4.751, *p* = 0.012, η*_*p*_*^2^ = 0.120], with amplitudes becoming less positive as a function of memory load (linear trends: posterior [*F*_(1, 35)_ = 66.306, *p* < 0.001, η*_*p*_*^2^ = 0.655]; anterior [*F*_(1, 35)_ = 6.180, *p* = 0.018, η*_*p*_*^2^ = 0.150]).

In the **older group**, only a significant main effect of Load emerged [*F*_(2, 68)_ = 32.980, *p* < 0.001, η*_*p*_*^2^ = 0.492], with EEG responses exhibiting more negative amplitude as load increased [quadratic trend: *F*_(1, 34)_ = 8.618, *p* = 0.006, η*_*p*_*^2^ = 0.202]^1^.

Conditioned on the results obtained on the P300 for the two groups, as a follow-up analysis we tested for a difference in load modulation (Load1—Load4 difference) between younger and older participants in both ROIs (regardless of hemisphere). The difference was significant (Bonferroni corrected alpha level: 0.05/2 = 0.025) for anterior channels [*t*(69) = −3.637, *p* = 0.001, *d* = 0.799, 95% CI = (−1.753 −0.449)], but not in the posterior ROI (*p* = 0.240). In the P300 latency, older adults exhibited greater load-related modulation over the anterior part of the scalp.

**FIGURE 10:**
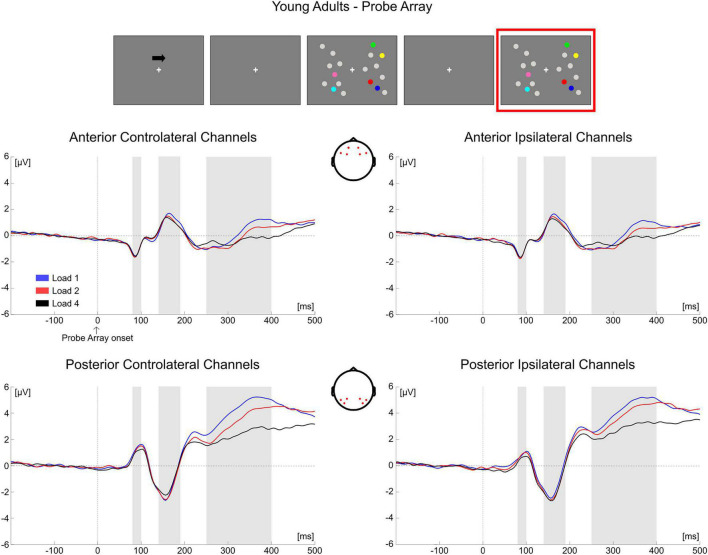
Grand average waveforms as a function of target load over the anterior (Fp1/2, F3/4, F7/8) and posterior ROI (P7/8, PO7/8, P3/4), separately for contra- and ipsilateral to target channels, in the Younger group during item comparison. Topographies highlight the channels included in the ROIs and gray squares indicate the TOIs over which the mean amplitudes were computed.

**FIGURE 11:**
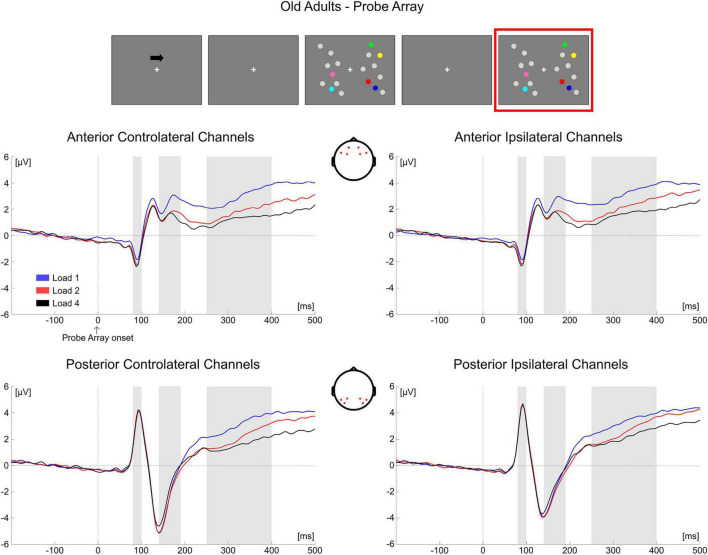
Grand average waveforms as a function of target load over the anterior (Fp1/2, F3/4, F7/8) and posterior ROI (P7/8, PO7/8, P3/4), separately for contra- and ipsilateral to target channels, in the Older group during item comparison. Topographies highlight the channels included in the ROIs and gray squares indicate the TOIs over which the mean amplitudes were computed.

As for the analysis of the memory array, these results are in line with the cluster-based analyses. During the presentation of the test array, younger subjects showed a modulation as a function of load only at later latencies (P300 time window) in both anterior and posterior ROIs, regardless of the hemisphere (Figure 10). In the older participants, ERPs were modulated by load at all the latencies that were considered, regardless of the hemisphere; such modulation was initially (P1 latency) evident for anterior channels, but then emerged also later in time (N1 and P300 latencies) in both anterior and posterior regions (Figure 11)^2^.

## Discussion

By exploiting a whole-scalp analysis approach we investigated the temporal development of age-related modifications in neural activity across and within the different processing stages of a task commonly used to assess the typical vWM capacity decrement of older adults (e.g., Jost et al., 2011; Sander et al., 2011; Störmer et al., 2013; Ko et al., 2014; Schwarzkopp et al., 2016; Tagliabue et al., 2019, 2020a). As expected (see Jenkins et al., 2000; Park et al., 2002), behavioral results showed a significant reduction in the older participants’ vWM capacity, even (slightly) at the lowest memory load. At the neural level, the results obtained from the whole-scalp approach (as well as the mean amplitude analysis) highlighted that in the group of older participants the load-related modulation encompassed several areas of the scalp, with supplementary activation from ipsilateral (during memory retention) and anterior sites (during both memory retention and comparison); in addition, the load effect was larger with respect to younger adults.

The present EEG findings go beyond the results obtained through the traditional EEG and fMRI approaches focusing on specific stages of stimulus processing and/or on specific brain areas (e.g., Grossman et al., 2002; Daffner et al., 2011), and further characterize the HAROLD and PASA accounts. Indeed, we did not focus on the typical response evaluated during item retention in previous ERP studies on age-related vWM decrement (namely, the CDA, e.g., Sander et al., 2011; Störmer et al., 2013). We rather investigated age-related brain dynamics (1) at a whole-scalp level and (2) during both item retention and retrieval, the latter being a processing stage that is usually not analyzed in DMTS tasks. Moreover, the choice of not selecting *a priori* TOIs was instrumental to observe the temporal dynamics of age-related modifications within and across stimulus processing stages.

### Item Retention Phase

A thorough analysis within this processing stage revealed that, during item retention (right after the offset of to-be-memorized items, from approximately 350 to 870 ms post-array onset), older adults showed a modulation as a function of load that overall encompassed posterior contralateral and ipsilateral electrodes, and bilateral anterior channels; in contrast, the effect was, at least initially, detectable only over posterior contralateral sites in the younger group. Although temporal and spatial inferences following cluster-based permutation tests should be made with caution (Sassenhagen and Draschkow, 2019), the time course of the neural activity provided by such analysis showed that in the older group the effect initially emerged over posterior contralateral channels, and then quickly broadened to include the whole scalp. Although the rapid spread of the effect over the whole scalp makes it difficult to clearly distinguish changes over the contralateral/ipsilateral and anterior/posterior axes, we speculate that the modulation as a function of load in ipsilateral channels is in line with the findings of age-related reduced processing asymmetry predicted by the HAROLD model (Cabeza, 2002): older adults’ neural activity was modulated by memory load not only over the “dominant” hemisphere (i.e., the contralateral hemisphere, as it would be expected given the lateralized target presentation), but also in the “non-dominant,” ipsilateral hemisphere^3^. Additionally, the whole-scalp analysis evidenced that this HAROLD-like modulation was dynamic, and emerged after an initial asymmetric engagement of posterior contralateral areas (similar to what occurred in younger adults). Finally, the bilateral activation was found over both anterior and posterior sites. While the initial formulation of the HAROLD model (Cabeza, 2002) referred to an age-related reduced hemispheric asymmetry mainly occurring in prefrontal areas, later work (including EEG studies) provided evidence in favor of such asymmetry reduction across several tasks and in more posterior regions (Grady et al., 2000, 2002; Nielson et al., 2002; Berlingeri et al., 2010; Learmonth et al., 2017).

The pattern of load-related modulations during item retention also complies with the PASA account (Davis et al., 2008). Indeed, the temporal course of the effect in older adults showed that, before encompassing all the channels bilaterally, it seemed to first develop toward contralateral frontal sites. This effect is in line with the increase in frontal activity claimed by the model for aging individuals, as it was present over frontal channels in the older group, but not in younger participants. We additionally observed that older adults showed a larger load-related modulation in posterior (contralateral) regions with respect to younger participants. Whereas the PASA initially predicted that the frontal over-recruitment is coupled to weaker activity of occipitotemporal areas in aging (Grady et al., 1994), such differential activation is not always found (e.g., Ansado et al., 2013; Morcom and Henson, 2018; notably, in this latter work, age-related increased activity over more posterior areas was evident). To account for such activation patterns, it was proposed (Ansado et al., 2013) that the PASA phenomenon is reflected more by an enhancement of frontal activity, rather than a clear dichotomy calling for reduced posterior and concurrent increased anterior cortical recruitment. Finally, traditional ERP analyses in this processing stage produced findings convergent with that of the cluster-based approach, namely load-related amplitude modulations evident in both ipsilateral and frontal ROIs in older adults.

In sum, the present data illustrate that the neural patterns of age-related activity during item retention are in agreement with the age-related activations predicted by both models, thus confirming the literature supporting their complementarity (see Festini et al., 2018 for a review). Specifically, at least initially we qualitatively observed no age effect on load-related modulations, since these appeared constrained to posterior contralateral channels as in younger adults. However, shortly afterward, both a shift of the load effect toward frontal areas (PASA) and a reduction in hemispheric asymmetry (HAROLD) were evident. This temporal pattern might suggest that the different age-related neural modifications are not a phenomenon indiscriminately present from the very beginning of stimulus processing, but could emerge over time.

In the younger group, only toward the end of the retention period (∼836–1,200 ms) a significant load effect emerged at posterior ipsilateral sites, and later included more central (but not anterior) electrodes. We speculate that two accounts may explain this late effect. First, due to the fixed temporal structure of a trial (there were no random temporal jitters), the effect may reflect anticipatory activity: younger adults might have refreshed the memoranda right before probe onset, as they became proficient in anticipating the sequence of the events after repeated presentations. This preparatory activity might have engaged also ipsilateral channels. Alternatively, the activity may reflect an attempt to suppress reorienting to the previously unattended side (see Rihs et al., 2009), scaled with the amount of cognitive engagement requested by the memorization of the different memory loads.

### Item Comparison Phase

During the presentation of the test array, results for both age groups indicated that load processing was evident bilaterally, suggesting that comparing the memory representation with the probe on screen is a more complex operation than the mere retention of items in the memory buffer. Several pieces of evidence have, indeed, been collected on interhemispheric cooperation when facing more complex cognitive tasks (e.g., Banich and Belger, 1990; Weissman and Banich, 2000). The results of whole-scalp and mean amplitude analyses revealed that older participants exhibited a load effect shortly after probe presentation. The effect was at first (around the P1 latency) over anterior areas, and later encompassed the posterior channels (from the N1 latency onward). In younger individuals, load-related modulations occurred later (around the P300 latency) and over posterior areas, then spreading to the whole scalp. The effect due to load processing was larger in frontal sites for the older adults, while it was of equal magnitude between the two groups in posterior areas (see also Ansado et al., 2013; Morcom and Henson, 2018). As above-mentioned for the item retention phase, the main feature of PASA-like activity seems to be a greater reliance on prefrontal activity in aging (Ansado et al., 2013). Therefore, the pattern of age-related differences observed during item comparison appeared consistent with the PASA view. Moreover, the load-related effect seemed to be anticipated (during P1 and N1 latencies) in aging, suggesting a greater engagement (i.e., more top-down control; Madden, 2007) in the initial perceptual and attentional processing stages.

### Comparison Between Groups

The direct comparison between groups (during both item retention and retrieval) in the whole-scalp analysis found significant differences only over specific time windows, and not over the entire segments that were analyzed. This, in turn, suggests that the observed inter- and intra-hemispheric remodeling was (at least) not entirely driven by age-related physiological changes (e.g., cortical or skull thickness, brain volume; Raz, 2000) that could induce different spreading gradients of the EEG signal in the two age groups. Future research will investigate the extent to which these age-related changes reflect the use of different cognitive strategies or modifications of the underlying brain networks (or both).

Some final issues should be discussed before conclusion. First, cluster-based permutation tests have some limitations in the temporal and spatial precision of the observed effects (Sassenhagen and Draschkow, 2019). Specifically, in older adults we observed that the presence of the load effect was rapidly evident over the whole-scalp, thus making it difficult to clearly discriminate ipsilateral vs. contralateral or posterior vs. anterior activity patterns. However, the absence of such distinct activity might be indicative of the complementarity of the PASA and HAROLD models, and also be in line with increasing evidence of reduced modularity in aging (Song et al., 2014; Geerligs et al., 2015; Knyazev et al., 2015). Indeed, the aging brain is characterized by a larger amount of random connections, thus resulting in a less segregated network structure (i.e., lower modularity; Gaál et al., 2010; Zangrossi et al., 2021). Since higher modularity is associated with higher cognitive performance (e.g., Baniqued et al., 2018), age-related reduced network segregation has been deemed (at least partially) responsible for the deficits in cognitive performance typically observed in aging (Zangrossi et al., 2021).

A second issue concerns the Compensation-Related Utilization of Neural Circuits Hypothesis (CRUNCH; Reuter-Lorenz and Cappell, 2008; see Jamadar, 2020 for a recent systematic review). This model states that the direction of the neural activation measured in the old age varies as a function of cognitive demands. More specifically, at lower cognitive loads older individuals tend to over-activate certain brain areas, leading to a good level of performance. However, they reach an activation plateau (the so-called “crunch point”) earlier than younger individuals, so that no further increase in neural activity is possible when task demands become higher, ultimately resulting in a concurrent decrement of performance. Here, we decided not to investigate such model since, in contrast with the HAROLD and the PASA accounts, CRUNCH does not hypothesize where in the brain age- or load-related changes might occur, making it difficult to build specific predictions in the current study.

To conclude, the current findings indicated that in a typical lateralized DMTS task older adults show broader brain engagement encompassing both inter- and intra-hemispheric sites. The findings are consistent with both HAROLD- (reduced hemispheric asymmetry) and PASA-like (anterior shift) activations. Noteworthy, such age-related activity changes manifest themselves in a dynamic way, as they vary at different stages of stimulus processing and progressively emerge over time.

## Data Availability Statement

The raw data supporting the conclusions of this article will be made available by the authors, without undue reservation.

## Ethics Statement

The studies involving human participants were reviewed and approved by the Comitato Etico per la ricerca—Università degli Studi di Trento. The patients/participants provided their written informed consent to participate in this study.

## Author Contributions

VM and CT developed the study concept and design. CT collected the data. GV and CT performed the data analysis and interpretation under the supervision of VM. All authors drafted the manuscript and approved the final version of the manuscript for submission.

## Conflict of Interest

The authors declare that the research was conducted in the absence of any commercial or financial relationships that could be construed as a potential conflict of interest.

## Publisher’s Note

All claims expressed in this article are solely those of the authors and do not necessarily represent those of their affiliated organizations, or those of the publisher, the editors and the reviewers. Any product that may be evaluated in this article, or claim that may be made by its manufacturer, is not guaranteed or endorsed by the publisher.

## Footnotes

1 The polynomial contrasts on performance (k) and EEG indexes (for both retention and retrieval) did not always show consistent load-related trends (linear or quadratic) between behavior and neural effects. This is reasonably due to the fact that the behavioral outcome only reflects the final output of different processing stages.

2 Failure to find a significant difference in the load effect between the two groups over earlier time points (i.e., corresponding to the traditional P1 and N1 latencies) in the cluster-based independent-samples *t*-test (see Figure 9) might be due to a decrease in sensitivity of this statistical test when comparing short-lasting ERPs.

3 Since the variation in the number of colored items presented in the relevant hemifield was independent from that of the irrelevant hemifield (see Stimuli and Procedure), we can associate the neural modulation observed with the number of targets appearing in the cued/relevant hemifield, and not with the elements in the uncued/irrelevant hemifield.
