# Supplementary material for: Inter- and Intra-Hemispheric Age-Related Remodeling in Visuo-Spatial Working Memory
Source: Front Aging Neurosci. 2022 Jan 17;13:807907. doi: 10.3389/fnagi.2021.807907 (PMC8803153; doi:10.3389/fnagi.2021.807907)
Supplement: Supplementary file 1 [file Data_Sheet_1.PDF]

Inter- and Intra-Hemispheric Age-Related Remodeling in Visuo-Spatial Working Memory

Chiara F. Tagliabue[1]*, Greta Varesio[1], Veronica Mazza[1]

[1]Center for Mind/Brain Sciences (CIMeC) – University of Trento

*** Corresponding Author**

Chiara F. Tagliabue

chiara.tagliabue@unitn.it

Center for Mind/Brain Sciences (CIMeC) – University of Trento

Corso Bettini 31, 38068 Rovereto (TN), Italy

	K values					
	Load 1		Load 2		Load 4	
	Young	Old	Young	Old	Young	Old
**Mean**	0.966	0.943	1.912	1.736	3.044	1.661
**Std. deviation**	0.037	0.039	0.084	0.169	0.551	0.559
**95% CI for mean (lower bound)**	0.966	0.930	1.884	1.678	2.857	1.469
**95% CI for mean (upper bound)**	0.979	0.956	1.941	1.794	3.230	1.853

Supplementary Table 1. Descriptive statistics of the behavioral variables (k values). (CI = confidence interval)

	CDA											
	Load 1				Load 2				Load 4			
	Posterior ROI				Posterior ROI				Posterior ROI			
	Contralateral		Ipsilateral		Contralateral		Ipsilateral		Contralateral		Ipsilateral	
	Young	Old	Young	Old	Young	Old	Young	Old	Young	Old	Young	Old
**Mean**	0.864	1.942	0.882	2.357	0.745	1.308	1.194	2.148	0.424	0.806	1.198	1.809
**Std. deviation**	1.751	2.060	1.618	1.963	1.744	1.942	1.548	1.880	1.921	2.051	1.759	1.918
**95% CI for mean (lower bound)**	0.272	1.234	0.334	1.683	0.155	0.641	0.670	1.502	-0.226	0.101	0.603	1.150
**95% CI for mean (upper bound)**	1.457	2.650	1.429	3.032	1.335	1.976	1.718	2.794	1.074	1.510	1.793	2.468
	Anterior ROI				Anterior ROI				Anterior ROI			
	Contralateral		Ipsilateral		Contralateral		Ipsilateral		Contralateral		Ipsilateral	
	Young	Old	Young	Old	Young	Old	Young	Old	Young	Old	Young	Old
**Mean**	-0.835	2.083	-0.778	2.532	-0.901	1.717	-0.803	2.573	-0.891	1.297	-0.768	2.213
**Std. deviation**	2.025	2.750	2.127	2.642	2.049	2.475	2.098	2.478	2.151	2.499	2.278	2.525
**95% CI for mean (lower bound)**	-1.520	1.139	-1.497	1.625	-1.594	0.867	-1.513	1.722	-1.618	0.438	-1.539	1.346
**95% CI for mean (upper bound)**	-0.150	3.028	-0.058	3.440	-0.208	2.567	-0.093	3.424	-0.163	2.155	0.003	3.081

Supplementary Table 2. Descriptive statistics of the EEG variables during item retention. (CI = confidence interval)

	80 – 100 ms (P100)											
	Load 1				Load 2				Load 4			
	Posterior ROI				Posterior ROI				Posterior ROI			
	Contralateral		Ipsilateral		Contralateral		Ipsilateral		Contralateral		Ipsilateral	
	Young	Old	Young	Old	Young	Old	Young	Old	Young	Old	Young	Old
**Mean**	1.317	3.211	0.810	3.658	1.184	3.114	0.807	3.630	1.041	3.243	0.572	3.701
**Std. deviation**	1.594	2.261	1.496	2.687	1.638	2.252	1.486	2.685	1.659	2.367	1.388	2.823
**95% CI for mean (lower bound)**	0.778	2.434	0.304	2.735	0.630	2.340	0.304	2.707	0.479	2.430	0.102	2.731
**95% CI for mean (upper bound)**	1.857	3.987	1.317	4.581	1.739	3.888	1.309	4.552	1.602	4.056	1.042	4.671
	Anterior ROI				Anterior ROI				Anterior ROI			
	Contralateral		Ipsilateral		Contralateral		Ipsilateral		Contralateral		Ipsilateral	
	Young	Old	Young	Old	Young	Old	Young	Old	Young	Old	Young	Old
**Mean**	-1.202	-1.286	-1.307	-1.356	-1.266	-1.655	-1.374	-1.644	-1.254	-1.802	-1.322	-1.798
**Std. deviation**	1.496	1.889	1.470	1.782	1.378	2.060	1.460	2.103	1.515	2.119	1.502	2.137
**95% CI for mean (lower bound)**	-1.708	-1.935	-1.804	-1.968	-1.732	-2.363	-1.868	-2.366	-1.767	-2.530	-1.831	-2.532
**95% CI for mean (upper bound)**	-0.696	-0.637	-0.809	-0.744	-0.800	-0.948	-0.881	-0.922	-0.742	-1.074	-0.814	-1.064
	140 – 190 ms (N100)											
	Posterior ROI				Posterior ROI				Posterior ROI			
	Contralateral		Ipsilateral		Contralateral		Ipsilateral		Contralateral		Ipsilateral	
	Young	Old	Young	Old	Young	Old	Young	Old	Young	Old	Young	Old
**Mean**	-1.486	-2.415	-1.417	-1.925	-1.567	-2.507	-1.687	-2.136	-1.315	-1.969	-1.724	-1.684
**Std. deviation**	2.625	1.702	2.423	1.597	2.573	1.749	2.451	1.540	2.636	1.904	2.428	1.773
**95% CI for mean (lower bound)**	-2.374	-3.000	-2.237	-2.474	-2.437	-3.108	-2.516	-2.665	-2.207	-2.623	-2.545	-2.293
**95% CI for mean (upper bound)**	-0.598	-1.830	-0.597	-1.376	-0.696	-1.906	-0.857	-1.607	-0.423	-1.315	-0.902	-1.075
	Anterior ROI				Anterior ROI				Anterior ROI			
	Contralateral		Ipsilateral		Contralateral		Ipsilateral		Contralateral		Ipsilateral	
	Young	Old	Young	Old	Young	Old	Young	Old	Young	Old	Young	Old
**Mean**	1.172	2.478	1.145	2.466	1.001	1.559	0.989	1.630	0.987	1.352	0.903	1.444
**Std. deviation**	2.222	2.192	2.264	2.261	2.166	1.857	2.165	1.862	2.079	1.769	2.055	1.848
**95% CI for mean (lower bound)**	0.421	1.726	0.378	1.689	0.269	0.922	0.257	0.991	0.283	0.744	0.208	0.809
**95% CI for mean (upper bound)**	1.924	3.231	1.911	3.243	1.734	2.197	1.722	2.270	1.690	1.960	1.599	2.079
	250 – 400 ms (P300)											
	Posterior ROI				Posterior ROI				Posterior ROI			
	Contralateral		Ipsilateral		Contralateral		Ipsilateral		Contralateral		Ipsilateral	
	Young	Old	Young	Old	Young	Old	Young	Old	Young	Old	Young	Old
**Mean**	4.248	3.101	4.224	3.278	3.363	2.114	3.761	2.536	2.358	1.592	2.788	2.132
**Std. deviation**	2.608	2.915	2.518	2.928	2.691	2.660	2.639	2.634	2.626	2.791	2.565	2.807
**95% CI for mean (lower bound)**	3.366	2.099	3.372	2.272	2.452	1.200	2.868	1.631	1.470	0.633	1.920	1.168
**95% CI for mean (upper bound)**	5.130	4.102	5.076	4.284	4.273	3.028	4.653	3.441	3.247	2.551	3.656	3.097

	Anterior ROI				Anterior ROI				Anterior ROI			
	Contralateral		Ipsilateral		Contralateral		Ipsilateral		Contralateral		Ipsilateral	
	Young	Old	Young	Old	Young	Old	Young	Old	Young	Old	Young	Old
**Mean**	0.191	2.921	0.092	3.114	-0.197	1.754	-0.250	2.102	-0.375	1.237	-0.380	1.557
**Std. deviation**	2.483	2.842	2.464	2.893	2.667	2.462	2.692	2.431	2.618	2.474	2.643	2.466
**95% CI for mean (lower bound)**	-0.649	1.944	-0.741	2.120	-1.100	0.908	-1.161	1.267	-1.261	0.387	-1.274	0.710
**95% CI for mean (upper bound)**	1.032	3.897	0.926	4.107	0.705	2.599	0.660	2.937	0.511	2.087	0.515	2.404

Supplementary Table 3. Descriptive statistics of the EEG variables during item comparison. (CI = confidence interval)
