# Supplementary figures and images for: Inter- and Intra-Hemispheric Age-Related Remodeling in Visuo-Spatial Working Memory
Source: Front Aging Neurosci. 2022 Jan 17;13:807907. doi: 10.3389/fnagi.2021.807907 (PMC8803153; doi:10.3389/fnagi.2021.807907)

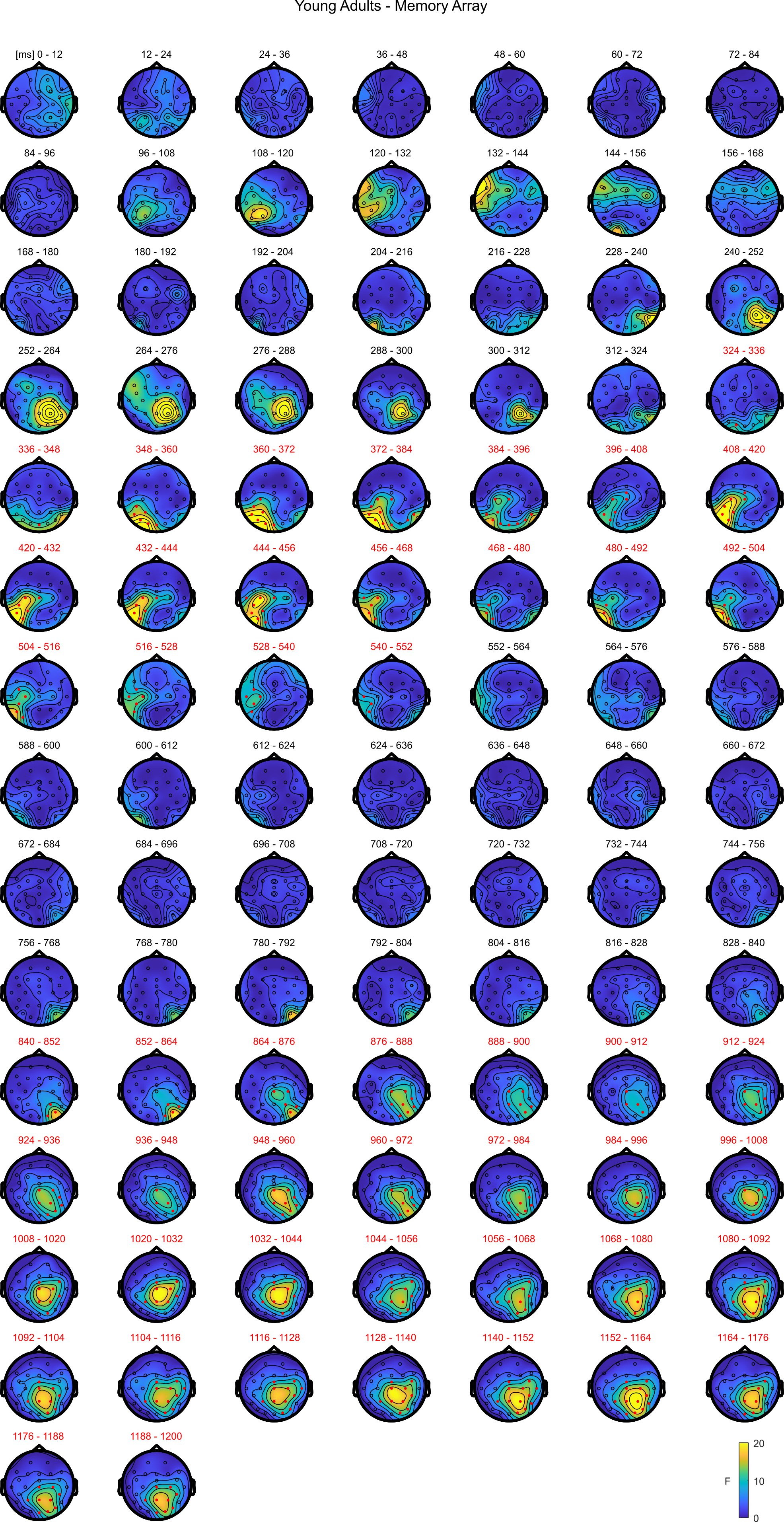

Supplement: Supplementary file 2 [file Image_1.TIFF]

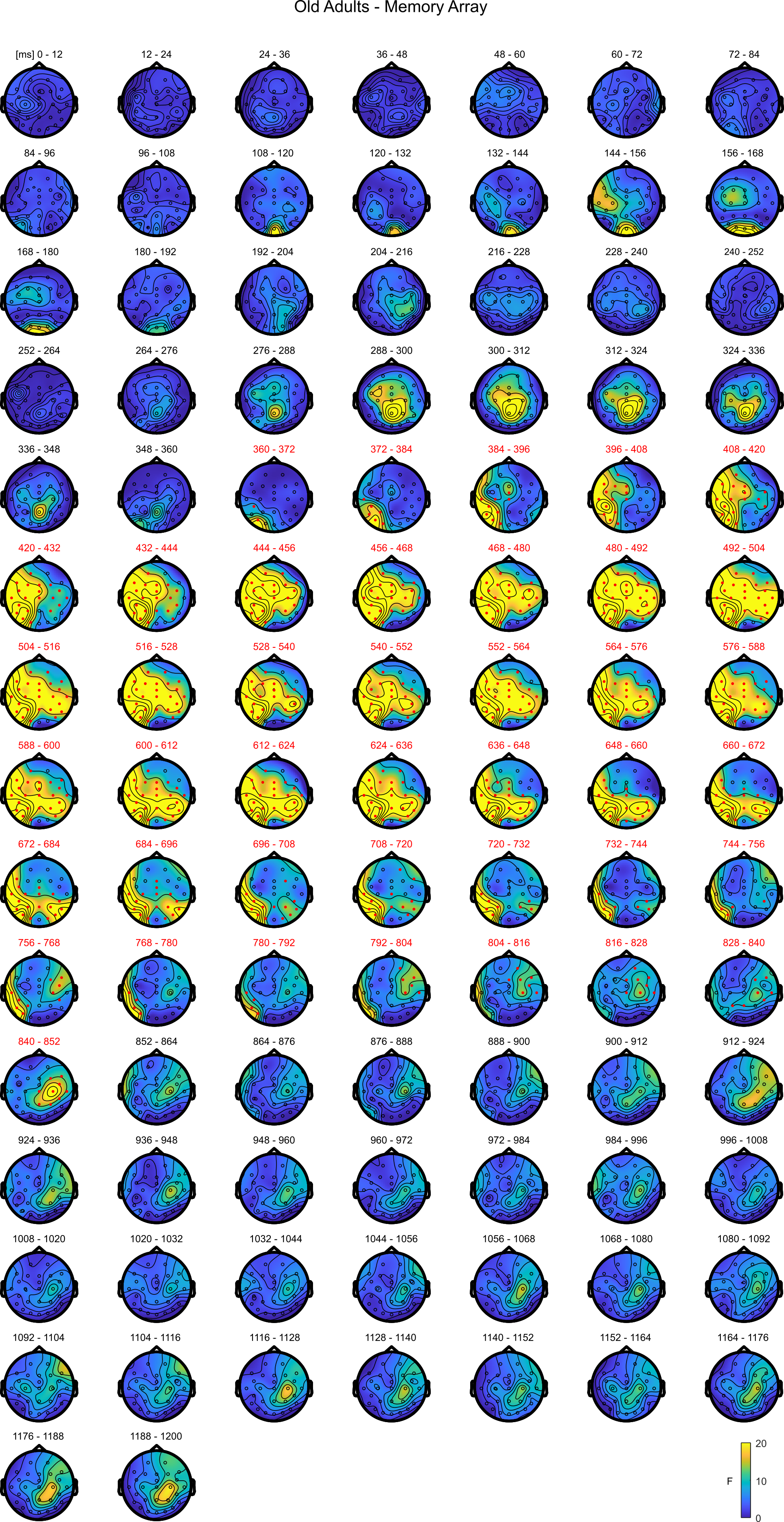

Supplement: Supplementary file 3 [file Image_2.TIFF]

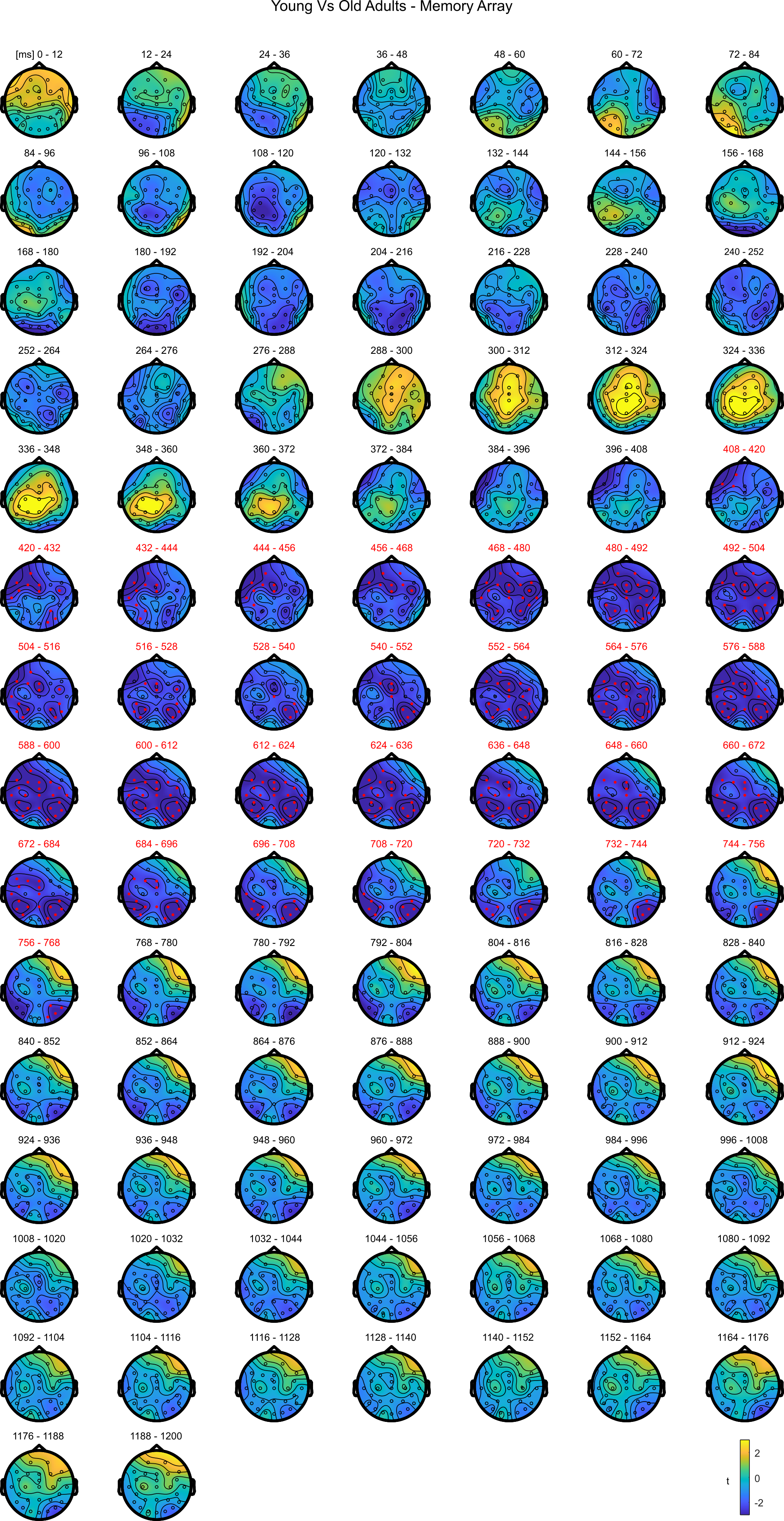

Supplement: Supplementary file 4 [file Image_3.TIFF]

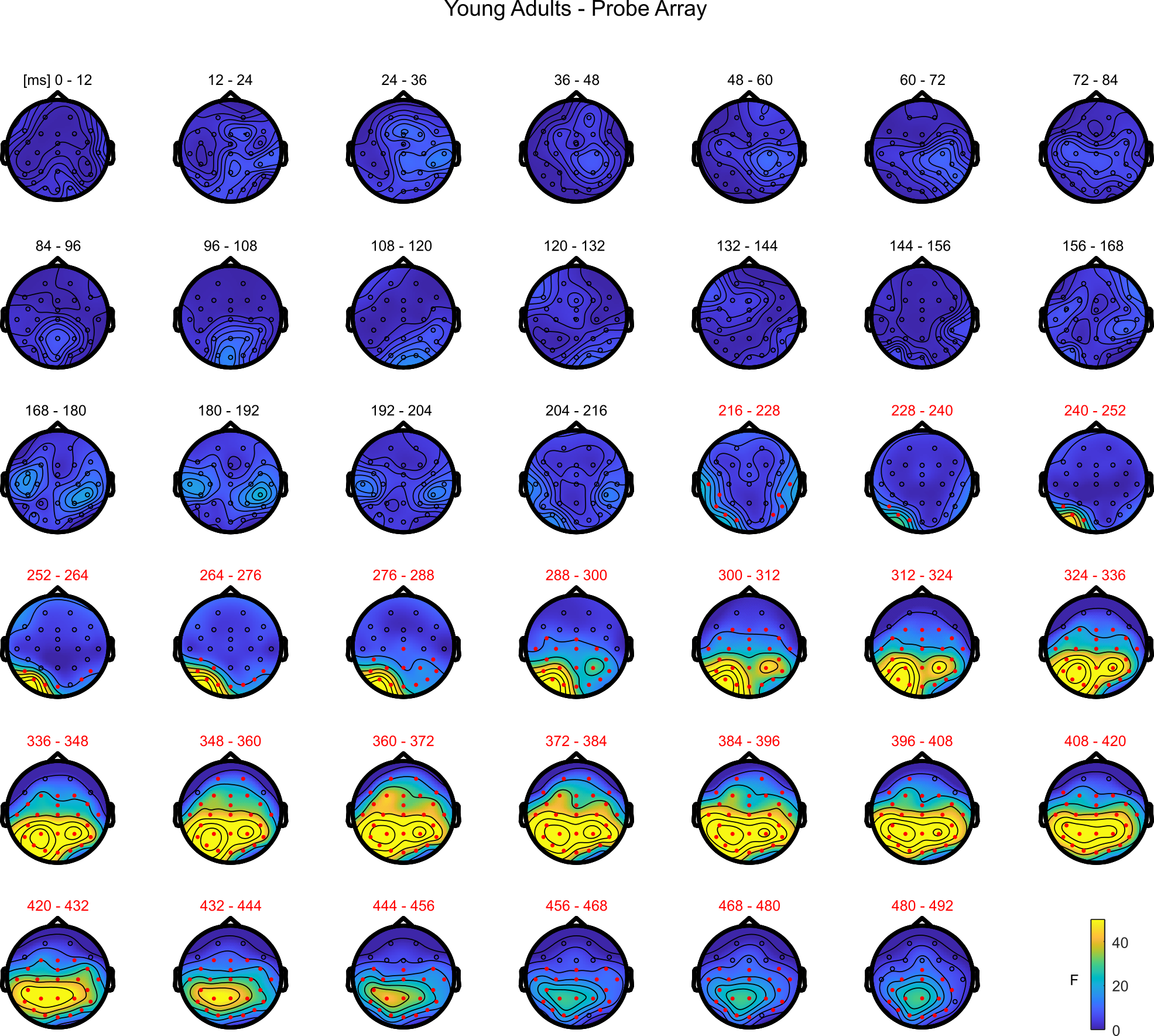

Supplement: Supplementary file 5 [file Image_4.TIFF]

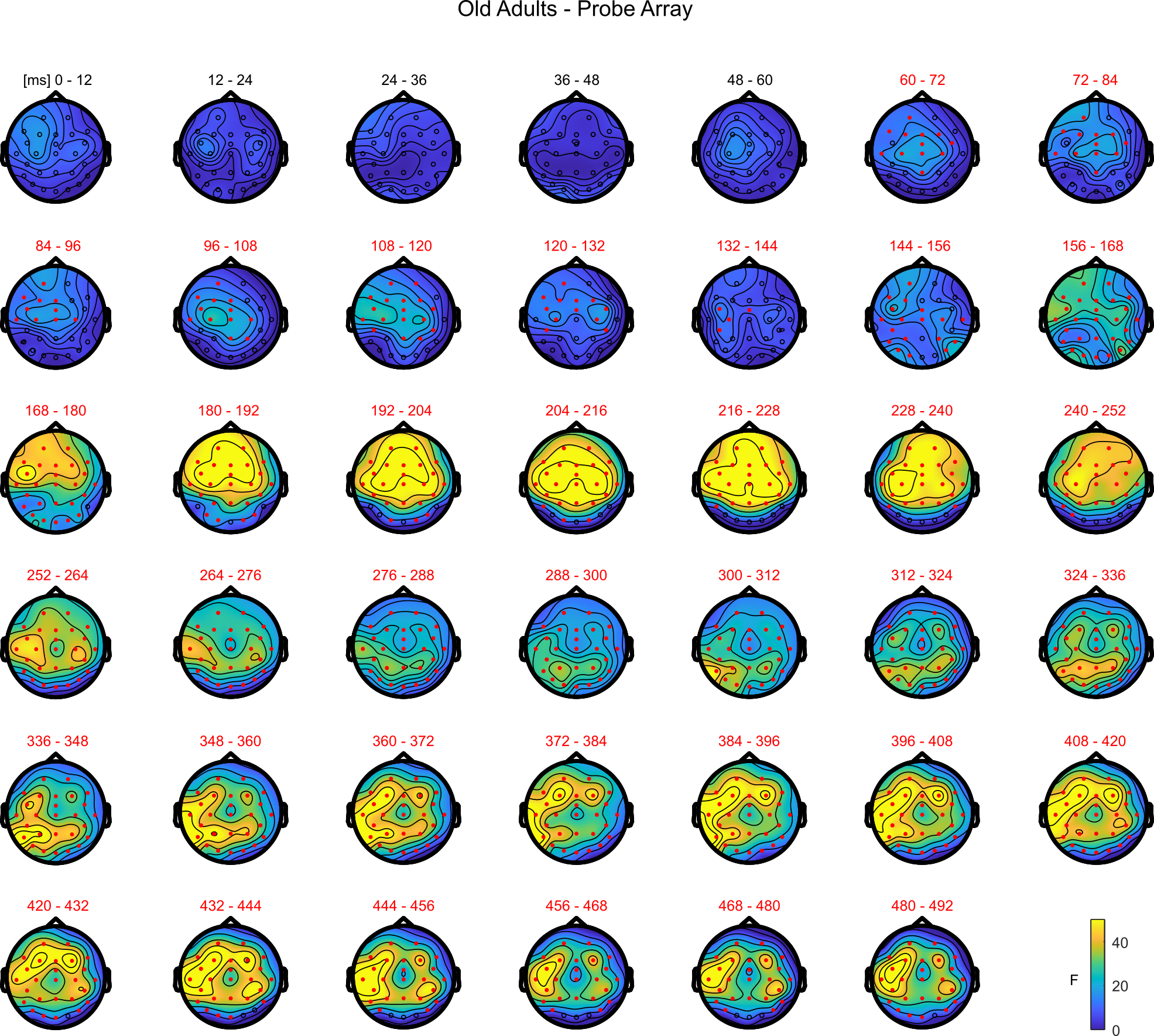

Supplement: Supplementary file 6 [file Image_5.TIFF]

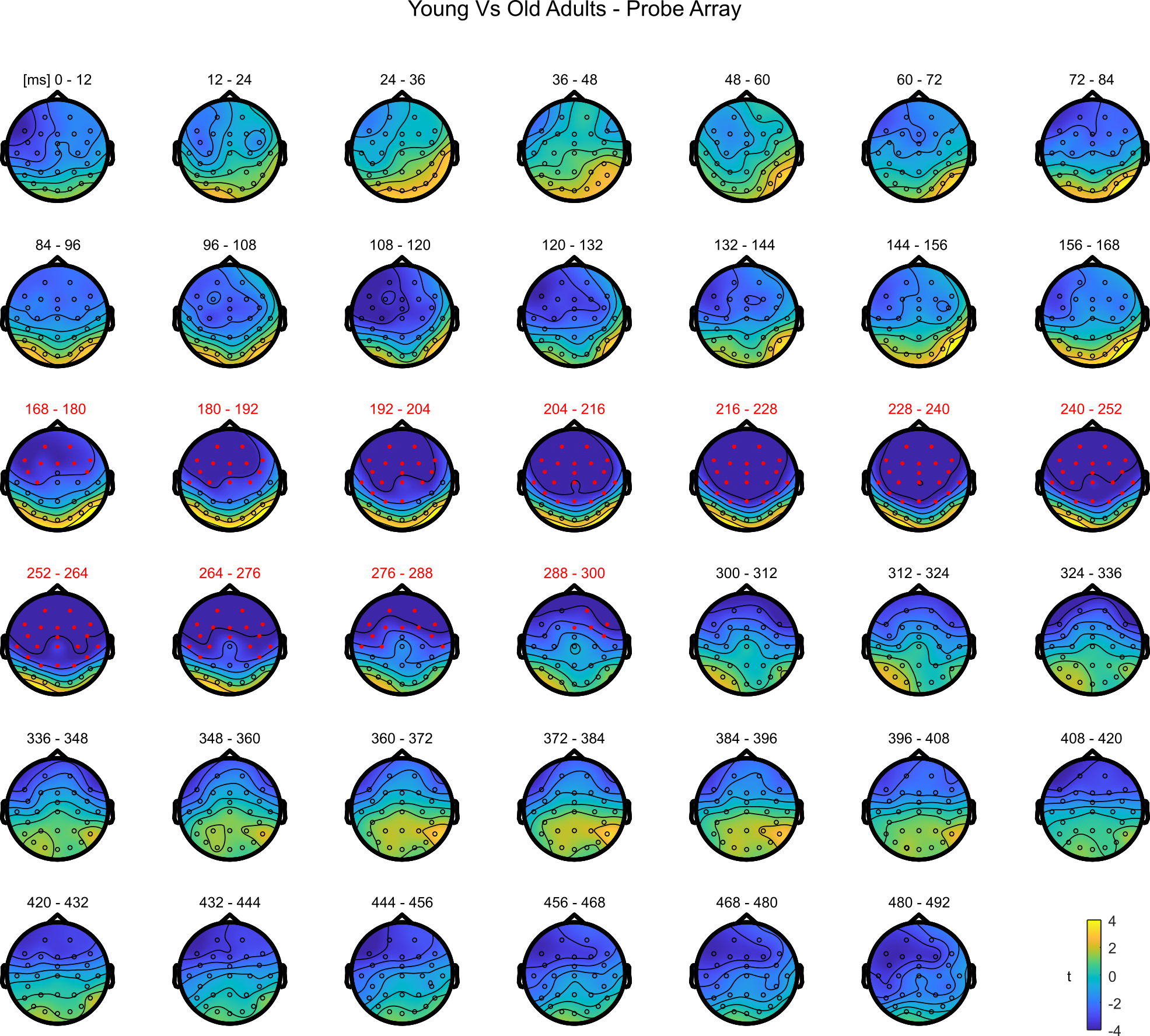

Supplement: Supplementary file 7 [file Image_6.TIFF]
